# Application of Machine Vision in Classifying Gait Frailty Among Older Adults

**DOI:** 10.3389/fnagi.2021.757823

**Published:** 2021-11-16

**Authors:** Yixin Liu, Xiaohai He, Renjie Wang, Qizhi Teng, Rui Hu, Linbo Qing, Zhengyong Wang, Xuan He, Biao Yin, Yi Mou, Yanping Du, Xinyi Li, Hui Wang, Xiaolei Liu, Lixing Zhou, Linghui Deng, Ziqi Xu, Chun Xiao, Meiling Ge, Xuelian Sun, Junshan Jiang, Jiaoyang Chen, Xinyi Lin, Ling Xia, Haoran Gong, Haopeng Yu, Birong Dong

**Affiliations:** ^1^National Clinical Research Center for Geriatrics, West China Hospital, Sichuan University, Chengdu, China; ^2^Geriatric Health Care and Medical Research Center, Sichuan University, Chengdu, China; ^3^Department of Geriatrics, West China Hospital, Sichuan University, Chengdu, China; ^4^College of Electronics and Information Engineering, Sichuan University, Chengdu, China; ^5^Department of Rehabilitation Medicine, West China Hospital, Sichuan University, Chengdu, China; ^6^Geroscience and Chronic Disease Department, The 8th Municipal Hospital for the People, Chengdu, China; ^7^Medical Examination Center, Aviation Industry Corporation of China 363 Hospital, Chengdu, China; ^8^West China School of Basic Medical Sciences and Forensic Medicine, Sichuan University, Chengdu, China; ^9^Medical College, Jiangsu University, Zhenjiang, China; ^10^Public Health Department, Chengdu Medical College, Chengdu, China; ^11^West China Biomedical Big Data Center, West China Hospital, Sichuan University, Chengdu, China; ^12^Med-X Center for Informatics, Sichuan University, Chengdu, China

**Keywords:** frailty, gait, machine vision, biomarkers, preventative health care, feature extraction

## Abstract

**Background:** Frail older adults have an increased risk of adverse health outcomes and premature death. They also exhibit altered gait characteristics in comparison with healthy individuals.

**Methods:** In this study, we created a Fried’s frailty phenotype (FFP) labelled casual walking video set of older adults based on the West China Health and Aging Trend study. A series of hyperparameters in machine vision models were evaluated for body key point extraction (AlphaPose), silhouette segmentation (Pose2Seg, DPose2Seg, and Mask R-CNN), gait feature extraction (Gaitset, LGaitset, and DGaitset), and feature classification (AlexNet and VGG16), and were highly optimised during analysis of gait sequences of the current dataset.

**Results:** The area under the curve (AUC) of the receiver operating characteristic (ROC) at the physical frailty state identification task for AlexNet was 0.851 (0.827–0.8747) and 0.901 (0.878–0.920) in macro and micro, respectively, and was 0.855 (0.834–0.877) and 0.905 (0.886–0.925) for VGG16 in macro and micro, respectively. Furthermore, this study presents the machine vision method equipped with better predictive performance globally than age and grip strength, as well as than 4-m-walking-time in healthy and pre-frailty classifying.

**Conclusion:** The gait analysis method in this article is unreported and provides promising original tool for frailty and pre-frailty screening with the characteristics of convenience, objectivity, rapidity, and non-contact. These methods can be extended to any gait-related disease identification processes, as well as in-home health monitoring.

## Introduction

Frailty is a state of increased vulnerability to stress, which may lead to a diminished homeostatic capacity across multiple physiological systems (Fried et al., 2009). Frail older adults are at an increased risk of premature death and various adverse health outcomes, including falls, fractures, disability, and dementia, all of which could result in a poor quality of life and an increased cost of healthcare resources, such as emergency department visits, hospitalisation, and institutionalisation (Kojima et al., 2019). The comprehensive geriatric assessment (CGA), which serves as the basis for geriatric medicine and research, is primarily aimed at identifying and quantifying frailty by examining various risk-prone domains and body functions (Lee et al., 2020). Fried’s frailty phenotype (FFP), the most acceptable face to face evaluation for frailty, includes five components, namely weakness, slowness, exhaustion, low physical activity, and unintentional weight loss; trained personnel takes up 20 min for one case (Fried et al., 2001). The Rockwood frailty index (RFI), involving 70 clinical deficits, which is usually generated from comprehensive health records, was less available for seniors with limited medical resources (Rockwood et al., 2005).

Human locomotion is a common daily activity and is also an acquired yet complex behaviour. It requires the involvement of the nervous system, many parts of the musculoskeletal apparatus, and the cardiorespiratory system (Adolph and Franchak, 2017). Individual gait patterns are influenced by age, personality, mood, and sociocultural factors. Some age-related neurological cases, such as sensory ataxia and Parkinson’s disorders, lead to unique gait disorders (Pirker and Katzenschlager, 2017). Furthermore, the preferred walking speed in older adults is a sensitive marker of general health and survival (Pirker and Katzenschlager, 2017).

However, recent researchers have focused on understanding the impact of the frailty state on various gait parameters beyond speed, because only the gait speed might not be sufficient to classify the frailty state of an individual. An improved classification can be achieved by referring to parameters such as the signal root mean square and total harmonic distortion instead of simply relying on the gait speed (Martinez-Ramirez et al., 2015). Previous studies have suggested that transitionally frail individuals exhibit a reduced locomotive speed, cadence, stride length, increased stride time, double support (as a percentage of the gait cycle), and stride time variability as compared to healthy individuals (Schwenk et al., 2014). Artificial neural networks might also help to further investigate the frailty of gait. Dawoon recently analysed the gait statistics gathered from gyroscopes placed on the feet using a long short-term memory network-based classifier (Jung et al., 2021). Akbari performed a Kinect-sensor machine learning methodology as a frailty classifier *via* functional assessment exercises including a walking test (Akbari et al., 2021).

Different approaches have been implemented to determine the gait characteristics in clinical research. Numerous studies were based on signals from floor sensors or wearable sensors, which can relatively provide precise time and space information. However, these accessory devices have limited their applicability, specifically in developing districts (Muro-de-la-Herran et al., 2014). Human gait recognition and behaviour understanding (GRBU), mostly without the use of contact sensors, has become a major research branch of machine vision using artificial neural network tools and has a wide range of applications in the field of anti-terrorism, intelligent monitoring, access control, criminal investigation, pedestrian behaviour analysis, reality mining, and medical care (Luo and Tjahjadi, 2020).

Gait recognition and behaviour understanding are primarily divided into data-driven (model-free) and knowledge-based (model-based) methods, based on the requirement of any relevant human pose parameters for feature extraction. The idea underlying model-based methods is the application of mathematical constructs to analyse walking movement as a representation of gait appearance using several ellipses or segments (Yoo et al., 2002). The main advantages of the model-based approach are that it can reliably handle occlusion (particularly human body self-occlusion), noise, scale, and rotation, as well as overcome poor robustness and its dependence on precise modelling of the human body. A model-free GRBU method extracts the statistical information of gait contours in a gait cycle and matches the contours reflecting the same shape and motion characteristics. A gait energy image (GEI) (Li et al., 2018), which is a classical representation of gait features, derives many energy images of related features, such as the frame difference energy images (Chen et al., 2009), gait optical flow images (Lam et al., 2011), and pose energy images (Roy et al., 2012). The advantages of these approaches are that: (1) they can obtain more comprehensive spatial information, focusing on each silhouette; and (2) they can gather more temporal information because specialised structures are utilised to extract sequential information. However, the GEI-like method requires a high computational power.

A huge demand for telemedical care emerged owing to the increasing spread of the corona virus disease 2019 (COVID-19), particularly among the older adults. The application of deep learning algorithms introduces real-time health analysis *via* video recording devices, such as a monitoring camera, web camera, or smartphones (Banik et al., 2021). A machine vision-based self-reporting method has the potential to significantly enhance accessibility and reduce costs in frailty evaluation. A deep learning gait assessment, conducted in a recent trial, based on a wearable sensor or force plates, is promising for disease screening and in-home monitoring (Zhang et al., 2020). However, older adults’ resistance to wearable devices and the additional cost of equipment might limit the scenarios of these technologies.

**FIGURE 1:**
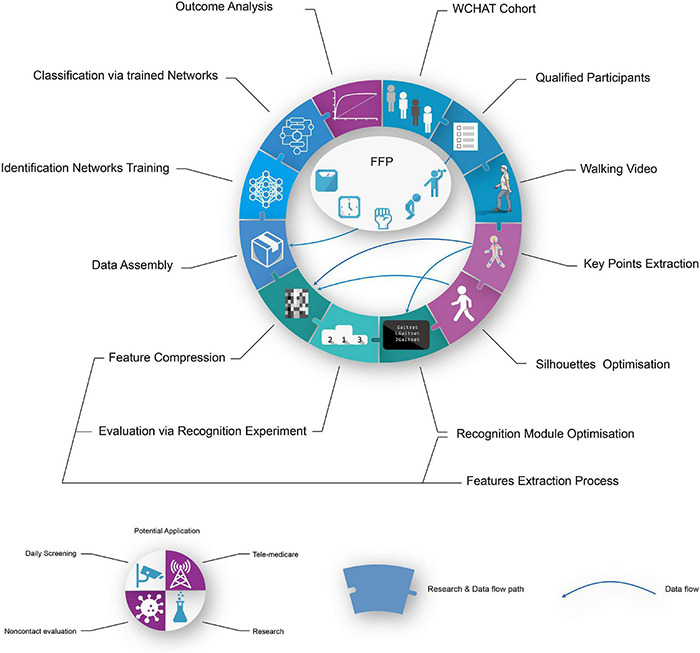
Visualised abstract. FFP, Fried Frail phenotype evaluation.

This study is aimed at developing a machine vision driven geriatric disease gait identification method without using a contact sensor or index and at exploring its potential as a frailty screening tool possessing the characteristics of convenience, objectivity, rapidity, and non-contact (Figure 1). In this study, a multidimensionally labelled gait video set of an older adult was established. Then, a series of hyperparameters in machine vision networks were optimised and evaluated for gait feature extraction and identification. The predictive power of the frailty and pre-frailty (patients at risk for frailty who fulfil some, but not all, criteria for frailty) evaluation was measured using the area under the curve (AUC) of the receiver operating characteristic (ROC). These methods may be generalised to any gait-related disease identification processes. The current approach provides unreported frailty and pre-frailty screening tools, which may potentially be generalised to gait-related diseases or in-home health monitoring, with the characteristics of convenience, objectivity, rapidity, and non-contact.

## Materials and Methods

### Participants

The current study involved a cross-sectional analysis of baseline data from the West China Health and Aging Trend (WCHAT) observational study that was designed to evaluate factors associated with healthy aging among community-dwelling adults aged 50 years and older in western China. From July to August 2019, we included a subset of 485 participants from five different locations in the Sichuan province. The final analysis consisted of 222 participants, excluding 205 individuals aged <60 years, 31 individuals who have difficulty in completing FFP evaluation safely, 24 individuals with a medical history of Parkinson’s disease or stroke (usually in a unique gait manner), and 3 individuals with incomplete walking video records. The recordings of participants who did not meet the criteria were used for an early-stage modification of the current pattern, such as the analysis of the body key points and a segmentation of the gait silhouettes. All participants (or their proxy respondents) were recruited by convenience, and they provided a written informed consent to the researchers, and our institutional ethics review boards approved the study. All researchers followed the local law and protocol to protect the rights of privacy, portraits, or other interests of the study participants.

### Frailty and Pre-frailty

Frailty and pre-frailty are defined using the FFP scale (Fried et al., 2001), comprising the following five elements: shrinking, slowness, weakness, exhaustion, and low physical activity. Subsequently, those who meet three or more of the above criteria are termed as frail, those who meet one or two are termed as pre-frail, and those who meet none of the criteria are called as non-frail or healthy older adults. In this study, a low physical activity was determined by the total amount of kcal/week spent on commonly performed physical activities as measured using a validated China Leisure Time Physical Activity Questionnaire (CLTPAQ) (Yanyan et al., 2019). Supplementary Table 1 presents more details on these criteria.

### Recording of Walking Video

**FIGURE 2:**
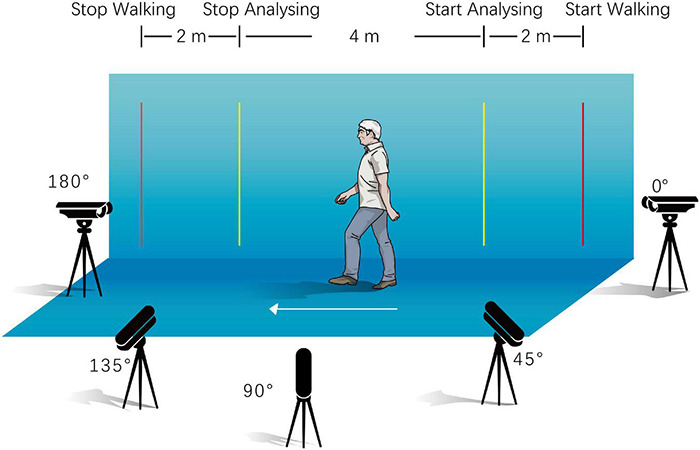
Recording of walking video.

Gait videos can be better shot in spacious, warm environments, on flat grounds, and in well-lit indoor environments. The green screen; two yellow parallel benchmarks, which were placed 4 m apart from each other; and five security cameras (*F* = 4 mm, DS-IPC-B12V2-I, Hikvision, Zhejiang, China) were properly fixed, as shown in Figure 2. The height of the cameras from the ground was approximately 1.3 m, and their angles were adjusted to ensure that the body of the entire gait process between the aforementioned benchmarks could be filmed and stored by the recorder (DS-7816N-R2/8P, Hikvision, Zhejiang, China) in an MP4 format at a 1080p resolution. Participants were requested to start walking 2 m ahead of the first benchmark and stop 2 m behind the second benchmark, at their usual speed. A complete recording of each participant included six 4 m-walking sequences and synchronised video segment shots from five different camera stands for each sequence, if possible. All the walking videos were manually edited, and only the footage consisting of the walking movement between the inner pair of benchmarks was collected for the end results. Subsequently, video files of every walking sequence were converted into frames of a static image.

### Machine Vision Approach and Analysis

There were two primary tasks underlying the gait identification process namely, gait feature extraction and feature classification. Feature extraction plays an important role in the identification and recognition processes, and directly affects their accuracy. Gaitset, a network that inputs a 64 × 64 walking silhouette sequence of a walking person recognition task, was used as a fundamental feature of the extraction network in the current study (Chao et al., 2021). In this study, the body key points were extracted before the silhouettes owing to their possession of different gait features, which were used as input for some silhouette segmentation modules. Then, the original silhouette segmentation and feature extraction methods were optimised and evaluated. Two classic pre-trained feature classifier networks were applied for the final frailty state identification.

#### Approach for Body Key Points Extraction

AlphaPose, an open-source pose estimation network, was used to extract the spatial information of the body key point from the original gait video (Fang et al., 2017). The performance of full-trained AlphaPose in key point extraction was evaluated by the quality of the merged image with the original image and the visualised body key point image in the current set. The framework of AlphaPose is presented in Supplementary Section 2 and Supplementary Figure 3. After evaluation, the body key point information of all walking image sequences was extracted *via* a pre-trained AlphaPose network. The operating system used was Ubuntu 16.04, and the graphics processing unit (GPU) was an NVIDIA GeForce GTX1080Ti graphics card. The trained model and setting were downloaded from GitHub and Google Drive (Supplementary Table 2).

#### Development and Treatment of Silhouettes Segmentation

**FIGURE 3:**
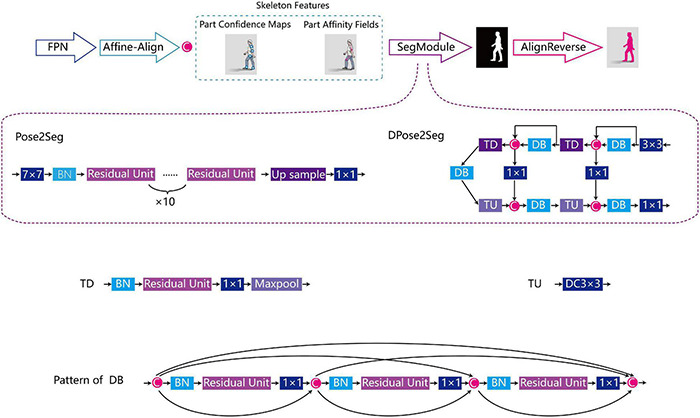
Frame structure of Pose2Seg and DPose2Seg. FPN, feature pyramid network; 7 × 7: 7 × 7 convolution kernel; 1 × 1: 1 × 1 convolution kernel; DC3 × 3: 3 × 3 deconvolution kernel; SegModule, segmentation module; BN, batch normalisation; DB, dense block; US, up sample; TD, transition down; TU, transition up; C, concat.

Pose2Seg (Zhang et al., 2019) is a posture-based approach to solve the segmentation problem of occluded human body instances (Figure 3). Firstly, the feature pyramid network (FPN) (Lin et al., 2017) extracts features from inputted standard image and the key point coordinates. After an affine-align operation based on human posture templates, two types of skeleton features are generated for each human instance, namely confidence maps and part confidence maps (Cao et al., 2017). The segmentation module is designed based on the same residual unit in Resnet (He et al., 2016). Finally, a reverse affine-align operation is performed on each instance to obtain the final segmentation results.

Our main optimisation of Pose2Seg was replace the original segmentation module with a module applicated fully convolutional DenseNets (Huang et al., 2017), and was named DPose2Seg. The concept of DenseNets is based on the observation that if each layer is directly connected to every other layer in a feed-forward fashion, the accuracy of the network will be improved (Jégou et al., 2017). We also experimented with another widely applied human body segmentation algorithm, Mask R-CNN (He et al., 2017).

The average precision (AP) (Yilmaz and Aslam, 2006), a pixel-level evaluation index in the image processing field, used the Mean value from 10 intersections over union thresholds, starting at 0.5 up to 0.95 with an interval of 0.05 steps. The mean average precision (mAP), which is defined as the AP values averaged over all the different classes, and with an AP larger than 96^2^ pixel (AP large) was used to measure the silhouette predictive power *via* the human body segmentation task in the opensource sets OCHuman (Zhang et al., 2019) and COCO2017 (COCO Consortium, 2017).

After evaluation, spatial information of the body key points and silhouettes of all packages were extracted and segmented by the best performance network in the trained status. The study environment for extraction and segmentation was as follows: the operating system used was Ubuntu 16.04, NVIDIA GeForce GTX1080Ti graphics card was the GPU, and PyTorch was tool programming the deep learning framework. The settings and parameters were as follows: 16 for batch size, 55 epochs for the entire training stage, 0.0002 for the initial learning rate, 0.00002 for the learning rate after the 33rd epoch, and the Adam algorithm was used to optimise the remaining parameters. The pre-trained Pose2Seg model (trained with COCO2017 and OCHuman) and setting files were downloaded from GitHub (Supplementary Table 2).

#### Development and Treatment of Gait Features Extraction

**FIGURE 4:**
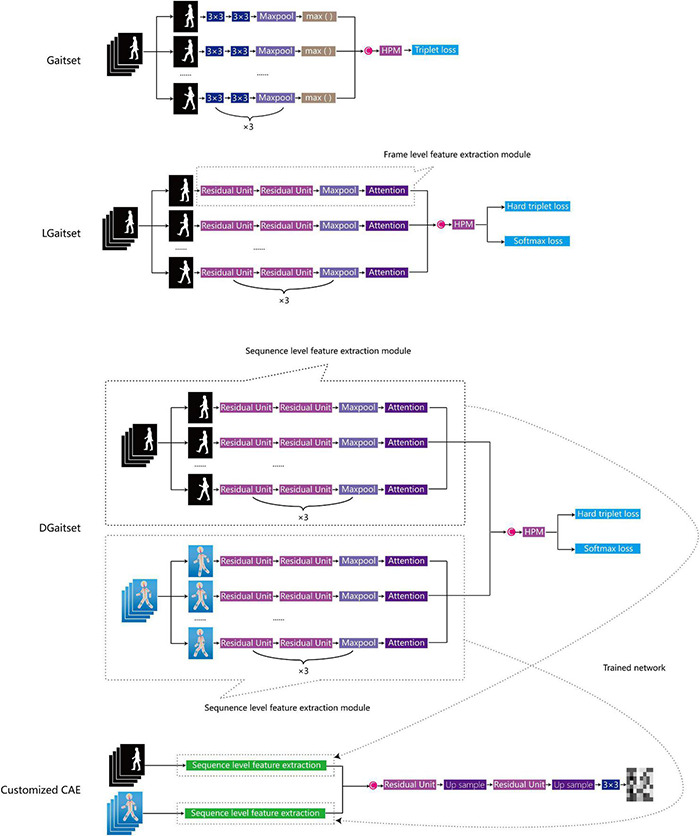
Overview of network structure of feature extraction methods. C, concat; HPM, horizontal pyramid mapping, attention: attention module, 3 × 3: 3 × 3 convolution kernel; CAE, convolutional auto-encoder.

In the training model of original Gaitset (Chao et al., 2019), a convolutional neural network (CNN) was used to extract frame-level features from each frame of the silhouette independently (Figure 4). Second, an operation called max pooling was performed to aggregate frame-level features. Third, after three repetitions of two previous steps and a concatenation of all frame-level outputs, a structure called horizontal pyramid mapping (HPM) (Fu et al., 2019) was implemented to map the sequence-level feature into a more discriminative space to obtain the final representation. Triplet loss was computed to the corresponding features among different samples and employed to train the network (Chao et al., 2021).

The first stage optimisation of Gaitset, named as LGaitset, included renewal of the loss function at the sequence level, and application of the attention module and residual units at the frame level (both were mentioned in the Gaitset study as alternative optimisations). To improve the convergence and performance of the model, LGaitset replaced triplet loss with the weighted sum of softmax loss and hard triplet loss (Taha et al., 2020). The attention module (Chao et al., 2019; Chaudhari et al., 2019) replaced the max function for an enhanced learning and the extraction of global features. The residual units partly replaced the convolution operation in the original network to improve the feature extraction ability and avoid the vanishing gradient problem (He et al., 2016; Chao et al., 2019).

The second-stage optimisation, DGaitset, a dual-channel input (silhouettes and body key point sequence) manner network structure based on LGaitset, was designed to achieve a better performance in feature extraction, focusing on the manner of imputation. Cause gait parameters, such as stride length, stride variation, and stride symmetry, are contained in the key point sequence and have been used as biomarkers in frailty evaluation.

The performance of these three methods was evaluated using an individual recognition task in the current walking dataset. The analysis set of the recognition task comprised data from 222 annotated individuals (1332 walking sequence) or from 6660 walking recordings, which were then shuffled and randomly separated into a 4/1 training/validation set split (Supplementary Figure 1). Recordings from an individual would enter in the training or validation set only, but not both sets to avoid a bias evaluation metrics. The validation set was maintained static during this experiment. Every gait recording in the validation set was input as a probe alternately to the three trained networks with a frozen weight and parameter; then, the remaining recording in validation would be regarded as a gallery and compared with the probe. In every epoch of the learning period, the system randomly split a few participants into the first four walking sequences, and the remaining recordings in the training set were regarded as the gallery set. The loss function’s objective was to calculate the distance from the probe recordings to a positive sample (belonging to the same individual) and a negative sample (not belonging to the same individual) in the gallery, and to adjust the wright and value in the model. A successful recognition refers to those recordings which exhibited the highest probabilities in the gallery, and belonged to the same individual who filmed the probe recording. The Chi-square test was conducted to test the significance of the recognition ratio between the models.

The hardware and software environments were similar to the previous silhouette sections. The settings and parameters of the Gait-set and LGaitset are batch size (8, 4), and every epoch takes 32 silhouette sequences (eight participants, four camera stands for every participant). The settings and parameters of DGaitset are batch size (6, 4), every training epoch takes 24 silhouettes and key point sequences (six participants, four camera stands for every participant), 80,000 epochs for the entire training stage, and 0.0001 for the initial learning rate. The Adam algorithm was used to optimise other parameters. Pre-trained Gaitset models were downloaded from the onedrive platform (Supplementary Table 2).

The sequence-level feature extraction part of the best performance-trained method was saved to generate a convolutional auto-encoder (CAE) (Masci et al., 2011) to compress the silhouettes and/or key point sequence into a 64 × 64 matrix for classification tasks in the next section.

#### Identification of Gait Features (Frailty State)

The project consists of two classic pre-trained image identification networks, AlexNet (Krizhevsky et al., 2017) and VGG16 (Simonyan and Zisserman, 2015), from the PyTorch platform for the purpose of gait feature classification (Supplementary Table 2). A three-class classification for frailty, pre-frailty, and health gait features was designed to evaluate the performance of AlexNet and VGG16 as frailty classifiers for the current dataset. The ground truth state for all gait features in this experiment was labelled using a previously performed FFP assessment.

The analysis set comprised data from 222 annotated individual or 6660 gait sequence features (64 × 64 resolution matrix) from each walking sequence, which were then shuffled and randomly separated into an 80/20% training and test set/validation set split. As depicted in Supplementary Figure 2, after the first split, the training and test sets contained 184 participants’ gait features, and the validation set contained 38 participants. The validation set was kept static throughout the experiment, and the testing set was used to evaluate the model performance at the end of each epoch during training and for hyperparameter optimisation. Data from the training and testing sets were randomly split in a 60/20% ratio by the units of sequence at the beginning of every training epoch to maximise the training effect. During the training process, the parameter of the lower convolutional layer was frozen. Only the last three fully connected neural layers of both AlexNet and VGG16 were customised. All weights of the identification model were frozen after 200 training epochs. The environment was similar to that described in the previous section. The settings and parameters of AlexNet and VGG16 are batch size 32 and 0.001 for the initial learning rate. The Adam algorithm was used to optimise the other parameters. The training epochs for AlexNet and VGG16 were 300 and 1000, respectively. The learning effect of the model during the training period was measured by calculating the accuracy in the test set and the loss function in the training set. The full-trained AlexNet and VGG16 output a series of probabilities for gait features in the validation set according to the classification of these features.

All details of the statistical analysis process were given in Supplementary Section 1.

## Results

### Characterisation of Participants in the Physical Frailty Status Subgroup and Analysing Set

We compared participants’ background information of the training and test set/validation sets (Table 1). We found no significant differences in age, gender, education level, marital status, and physical frailty status prevalence between the training/test and validation sets.

**TABLE 1:** Characterisation and physical status of participants among 222 older adults aged >60 in the three-class identification experiment.

**Characteristic**	**Prevalence, *n* (%)**	**Within physical frailty status, *n* (%)**			**Within analysing set, *n* (%)**	
		**Healthy**	**Pre-frailty**	**Frailty**	**Training and testing**	**Validation**
All participants	222	107 (48.2)	82 (36.9)	33 (14.9)	184 (82.9)	38 (17.1)
Age, years (Means ± SD)	68.9 ± 6.0	67.4 ± 5.0	69.2 ± 6.4	72.8 ± 6.3	69.0 ± 6.0	68.6 ± 6.2

**Gender**						

Male	117 (52.7)	61 (57.0)	41 (50.0)	15 (45.5)	98 (53.3)	19 (50.0)
Female	105 (47.3)	46 (43.0)	41 (50.0)	18 (54.5)	86 (46.7)	19 (50.0)

**Physical frailty status**						

Healthy	107 (48.2)	–	–	–	89 (48.4)	18 (47.4)
Pre-frailty	82 (37.0)	–	–	–	68 (37.0)	14 (36.8)
Frailty	33 (14.9)	–	–	–	27 (14.7)	6 (15.8)

**Education level**						

Primary or illiterate	169 (76.1)	78 (72.9)	60 (73.2)	31 (93.9)	142 (77.2)	27 (71.1)
Junior high	40 (18.0)	25 (23.4)	14 (17.1)	1 (3.0)	32 (17.4)	8 (21.1)
Senior high or higher	13 (5.9)	4 (3.7)	8 (9.8)	1 (3.0)	10 (5.4)	3 (7.9)

**Marital status**						

Married	171 (77.0)	90 (84.1)	60 (73.2)	21 (63.6)	146 (79.3)	25 (65.8)
Others	51 (23.0)	17 (15.9)	22 (26.8)	12 (36.4)	38 (20.7)	13 (34.2)

**Positive prevalence for frailty phenotype**						

Shrinking	12 (5.4)	0	6 (7.3)	6 (18.2)	8 (4.3)	4 (10.5)
Slowness	65 (29.3)	0	34 (41.5)	31 (93.9)	56 (30.4)	9 (23.7)
Weakness	57 (25.7)	0	28 (34.1)	29 (87.9)	44 (23.9)	13 (34.2)
Exhaustion	32 (14.4)	0	23 (28.0)	27 (81.1)	27 (14.7)	5 (13.2)
Low physical activity	50 (22.5)	0	23 (28.0)	27 (81.1)	43 (23.4)	7 (18.4)

**Body measurement (Means ± SD**)						

BMI, Kg/m^2^	25.3 ± 3.5	26.0 ± 3.1	25.2 ± 3.6	23.7 ± 4.3	25.3 ± 3.6	25.6 ± 3.4
Time of 4 m walking, s	7.3 ± 2.2	6.2 ± 0.7	7.4 ± 1.6	9.6 ± 3.3	7.3 ± 2.0	7.5 ± 3.2
Grip strength, Kg	26.9 ± 8.9	31.0 ± 8.0	25.2 ± 7.8	18.2 ± 6.2	27.2 ± 9.0	25.7 ± 8.5

### Body Key Points and Silhouettes

**FIGURE 5:**
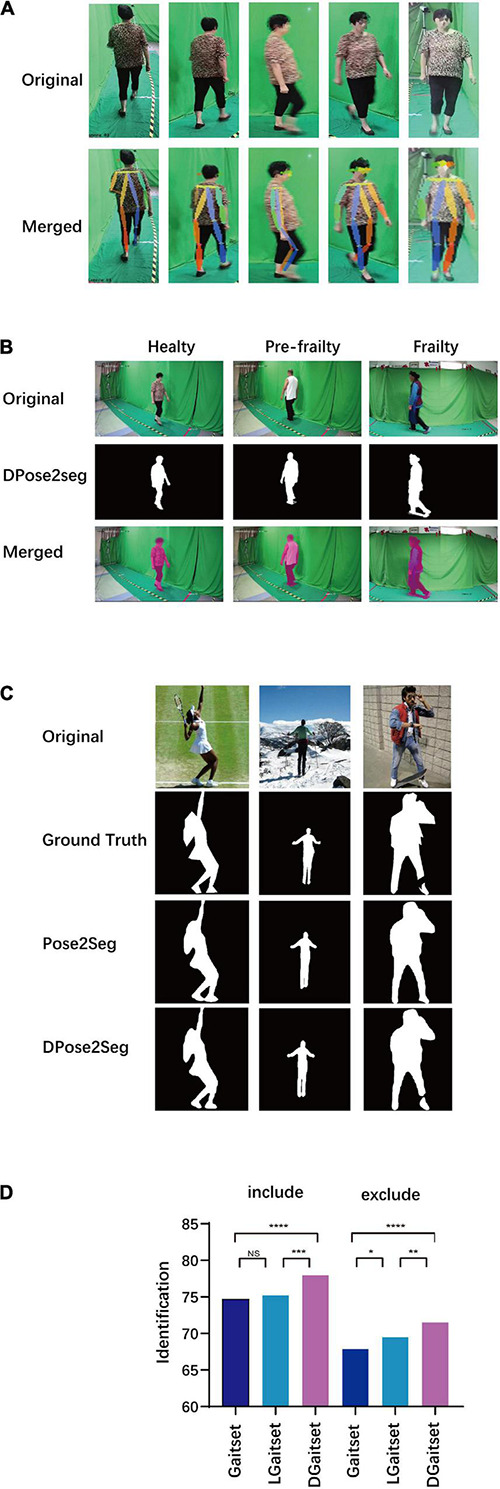
Results of key point extraction, silhouette segmentation, and gait feature extraction. **(A)** Sample of body key points extraction (original image and visualisation of key points parameters merged with original). **(B)** Sample of silhouette segmentation in current dataset. **(C)** Sample of silhouette segmentation in COCO dataset. **(D)** Results of Gait identification task *via* different method. Include: validation set includes the gait sequence filmed from the same camera stand with the probe. Exclude: validation set excluding gait sequence filmed from the same camera stand with probe; NS, *P*-value for difference of successful identification ratio between methods ≥0.05; *P < 0.05; **P < 0.01; ***P < 0.005; ****P < 0.001.

Figure 5A demonstrates the outcome of AlphaPose-applicated senior gait footage. The sample of merged images with the original image and key points in the current set evaluated by human vision presented satisfactory performance in body key point recognition for the current method. The body key point information or skeleton information, obtained as output from AlphaPose, was used as a part of the input in the segmentation and feature module customisation process.

Figures 5B,C and Supplementary Videos 1–6 presents the silhouette segmentation samples in the open dataset and target dataset *via* DPose2Seg and Pose2Seg. In the comparison of the precision of segmentation methods, DPose2Seg presents an advantage to Pose2Seg and Mask R-CNN in testing of labelled human image datasets, OCHuman and COCO2017 (Supplementary Table 3). Thus, trained DPose2Seg, though the current segmentation task, would generate the silhouettes needed in the following experiments.

The computational time consuming for different machine vision analysis task was presented in Supplementary Table 4.

### Reorganisation and Feature Extraction

A reorganisation comparison was conducted to examine the performance of deep-learning models with respect to the gait feature extraction in the target dataset (Figure 5D). DGaitset had both better included and excluded the same camera stand with a probe at the gallery in an individual reorganisation test than the LGaitset and original Gaitset methods. Thus, the sequence level feature extraction part of the CAE was customised as DGaitset trained in the current experiment. All gait features contained in the body key points and silhouette sequence were arranged in a 64 × 64 matrix *via* the CAE.

### Performance of Gait Classification

**FIGURE 6:**
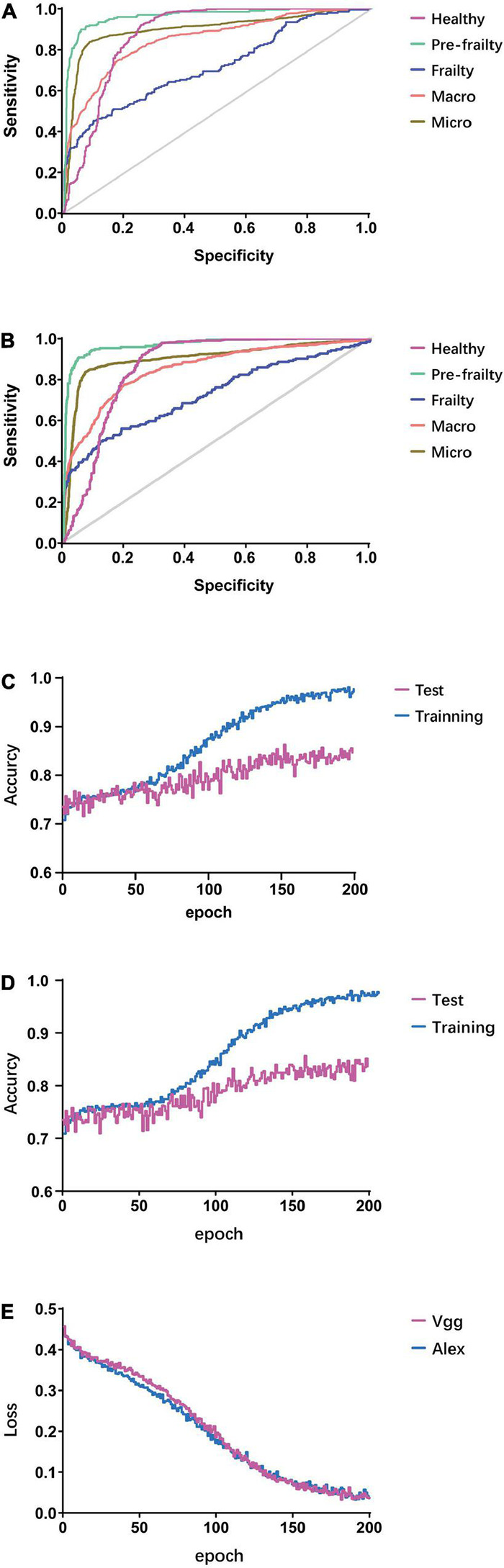
Results of gait feature classification. **(A)** ROCAUC of validation set *via* AlexNet. **(B)** ROCAUC of validation set *via* VGG. **(C)** Accuracy *via* AlexNet. **(D)** Accuracy *via* VGG16. **(E)** Loss function of training set.

In the three-class classification test, the AUC of ROC for AlexNet was 0.851 and 0.901 for macro and micro (Table 2 and Figures 6A,B), respectively, 0.872 for health state identification, 0.965 for pre-frailty identification, 0.715 for frailty; AUC of ROC for VGG16 was 0.855 and 0.905 for macro and micro, respectively, 0.866 for health state identification, 0.972 for pre-frailty, and 0.728 for frailty. The ROC AUC was found to be above 50% in all three classification tasks *via* AlexNet and VGG16 (all *P*-values < 0.0001).

**TABLE 2:** Predictive performance of physical frailty state classification *via* different method.

**Classification**	**Performance**	**Method**				
		**AlexNet**	**VGG16**	**Grip strength**	**4 m walking time**	**Age**
Global	ROC (macro)	0.851 (0.827–0.8747)****	0.855 (0.834–0.877)****	–	–	–
	ROC (micro)	0.901 (0.878–0.920)****	0.905 (0.886–0.925)****	–	–	–
	Kappa	0.636 (0.593–0.679)	0.659 (0.617–0.702)	–	–	–
	Accuracy	0.855	0.847	–	–	–

Healthy	ROC	0.872 (0.846–0.898)****	0.866 (0.831–0.889)****	0.754 (0.602–0.907)**	0.765 (0.615–0.916)***	0.629 (0.447–0.812) NS
	Sensitivity	98.40% (97.22–99.17%)	98.14% (96.89–98.98%)	–	–	–
	Specificity	63.85% (58.86–68.62%)	65.30% (60.33–70.02%)	–	–	–
	PPV	83.96% (82.10–85.66%)	84.52% (82.64–86.22%)	–	–	–
	NPV	95.40% (92.17–97.34%)	94.78% (91.48–96.84%)	–	–	–

Pre-frailty	ROC	0.965 (0.951–0.980)****	0.972 (0.961–0.987)****	0.695 (0.521–0.869)*	0.552 (0.365–0.739) NS	0.624 (0.433–0.814) NS
	Sensitivity	87.69% (82.24–91.95%)	89.80% (84.68–93.65%)	–	–	–
	Specificity	95.24% (93.68–96.51%)	95.87% (94.40–97.05%)	–	–	–
	PPV	79.17% (73.98–83.55%)	81.86% (76.78–86.03%)	–	–	–
	NPV	97.40% (96.27–98.20%)	97.84% (96.76–98.56%)	–	–	–

Frailty	ROC	0.715 (0.672–0.759)****	0.728 (0.677–0.773)****	0.635 (0.392–0.878) NS	0.906 (0.807–0.999)***	0.526 (0.262–0.790) NS
	Sensitivity	22.05% (16.44–28.53%)	25.91% (19.88–32.69%)	–	–	–
	Specificity	99.79% (99.24–99.97%)	99.68% (99.08–99.93%)	–	–	–
	PPV	95.56% (84.01–98.88%)	94.34% (84.01–98.14%)	–	–	–
	NPV	86.12% (85.20–86.99%)	86.84% (85.86–87.77%)	–	–	–

Kappa for kappa coefficients, PPV for positive predictive value, NPV for negative predictive value, ROC for area under the curve of the receiver operating characteristic, (95% CI), NS for *P* ≥ 0.05 for ROC AUC > 50%, *P < 0.05 for ROC AUC > 50%, **P < 0.01 for ROC AUC > 50%, ***P < 0.005 for ROC AUC > 50%, ****P < 0.0001 for ROC AUC > 50%.

The machine vision gait feature classification methods (AlexNet and VGG16) performed a non-inferiority physical frailty state prediction using characteristics comparable to the 4 m walking time, as well as a better prediction than those carried out by considering the participant’s age and grip strength characteristics. By converting the three-class classification task to three different binary-classification tasks, the AUC of ROC for grip strength, age, and 4 m walking time to predict the physical frailty state in participants of the validation set were calculated as a contrast. A 4 m walking time exhibited a better predictive power than other methods in frailty identification (0.906, 95% CI 0.807–0.999), but not in pre-frailty identification (0.552, 95% CI 0.365–0.739). However, both machine vision methods showed superior advantages in pre-frailty classification compared to other methods. Grip strength showed a significant predictive value in healthy identification. The age of participants did not present a significant predictive value in any of the three classifications (*P* > 0.05).

The accuracy in the initial training period for the test set of both models was around 0.71–0.72, and a high classification accuracy for the test set was achieved during the 152nd epoch for AlexNet (0.862) and 158th epoch for VGG16 (0.856) (Figures 6C,D). The loss function for both models was approximately 0.45 at the beginning of learning, and it was below 0.1 mostly after the 130th epoch for both methods. The lowest loss was 0.0332 and 0.0327 for VGG16 (182nd epoch) and AlexNet (191st epoch), respectively (Figure 6E).

## Discussion

In the current study, a machine vision method without using a contact sensor was implemented to identify frailty and pre-frailty among older adults based on their walking behaviour. First, an FFP-state-labelled senior walking video dataset consisting of 222 participants was created. All images of their gait sequences were treated using the body key point information extraction application AlphaPose. DPose2Seg, with a fully convolutional DenseNets segmentation module, also trained by an open-source human body image set, was the silhouette segmentation measure for the previous gait set. Gait body key points and silhouette information were used in a trained recognition network, DGaitset. The sequence-level feature extraction part in the trained DGaitset generated a customised CAE to compress the gait feature in a 64 × 64 matrix using the key points and silhouette sequences. We found that both machine vision methods (AlexNet and VGG16) equipped with better predictive performance globally than age and grip strength, as well as than 4-m-walking-time in healthy and pre-frailty classifying task.

In the pre-treatment stage, opensource AlphaPose and optimised Pose2Seg were used as tools for body key point extraction and silhouette segmentation. The first step of body key point extraction was the location of the human body, i.e., bounding the human within boxes. The inevitable small errors in body localisation can cause failures in a single-person body key point extraction. AlphaPose can handle inaccurate bounding boxes and redundant detections (Fang et al., 2017). The key point information output from the pre-trained AlphaPose not only provides mathematically constructed pose information to three silhouette segmentation methods but also provides part of the input for DGaitset and customisation of CAE. The replacement of layers of the residual unit with a fully convolutional DenseNets structure increased the precision of segmentation, which introduces the candidacy of DPose2Seg along with AlphaPose to function as a silhouette-generated method for other gait research.

As gait motion was a one-circle period, all silhouette clouds were represented in a single period. Our fundamental network, Gaitset, a model-free GRBU method, directly learns the representation of every frame silhouette independently *via* a CNN and set pooling instead of measuring the similarity between a pair of silhouette templates or sequences (Chao et al., 2019). Furthermore, a structure called HPM was used to map the set-level feature into a more discriminative space. Thereafter, the recognition was completed by calculating the distance between the representations of different samples. Gaitset exhibited a faster and more effective performance in individual re-identification tests in comparison with the previous model-free methods. The three major optimisations of DGaitset were as follows: replacement of max function with the attention module (in frame and sequence level), replacement triplet loss to weighted sum of softmax loss and hard triplet loss (in sequence level), and dual-channel input manner network (in global structure). DGaitset, a hybrid of model-based and model-free GRBU manner, performed better than the Gaitset and LGaitset approaches in the recognition task, and also suggests that DGaitset is a better candidate for the gait feature extraction and compression using the original video.

AlexNet used the residual unit activation function after the convolutional layers and softmax for the output layer, as well as applied max pooling instead of average pooling (Gu et al., 2018). VGG uses very small convolutional filters and very deep (16 and 19 layers) models (Bajić et al., 2019). The design decisions in the VGG models have become the starting point for the simple and direct use of CNNs in general.

The limitation of the current programme was the gait feature labelled by the FFP assessment excluding the prognosis events such as death, major cardiovascular events, or re-hospital, which directly point to a state of frailty because follow-up data of the WCHAT study is currently unavailable. The scale of the current walking video database and the unbalanced physical frailty state prevalence in the community-based cohort also limited the performance of the machine vision frailty classifier. As the potential clinical-gait-machine vision applications based on the current research may focus more on disease screening than accurate diagnosis, discarding random samples from the healthy group in the data compilation stage, or increasing the cost of the frailty group in the algorithm modification stage could increase the sensitivity to frailty state in the future development (Van Hulse et al., 2007; Krawczyk, 2016).

Although AlphaPose is a reliable method for building body key point images, its 17 key points (nose, left and right eyes, ears, shoulders, wrists, hips, knees, and ankles) did not include any points on the feet (Task Force Members et al., 2013). This deficit might cause an increase in noise around the feet compared with other body parts in silhouette segmentation. The latest body point extraction algorithm could label ankle, heel, and foot index, such as BlazePose and Zou’s method, which might provide a better choice for the machine vision body reconstruction model in this field (Bazarevsky et al., 2020; Zou et al., 2020). As most non-linear machine learning methods (Horst et al., 2019), part of the analysis processes in the current program were not straightforward, understandable, and interpretable.

Our methodology performs unique advantages in identifying the pre-frailty state, which might provide a clue for developing a novel biomarker. Pre-frailty is usually not as typical as frailty, which limits the proper preventive treatment, such as physical exercise, nutritional interventions, and implements (Serra-Prat et al., 2017). Furthermore, the current camera-based identification methods might extend their potential applications from frailty to other geriatric syndromes, such as cognitive impairment (Amboni et al., 2013).

A contact-free self-reported frailty assessment tool, based on this method, might help healthcare personnel (HCP) minimise their exposure to SARS-CoV-2-contaminated environment and equipment. It is well known that frailty status is a better predictor of prognosis than age in the COVID-19 therapy process (Hewitt et al., 2020). The evaluation of FFP depended on the face-to-face evaluation of HCPs, and HCP might not be able to enough detailed information within 30 min to make a comprehensive RFI evaluation for elderly patients diagnosed with hearing, visual or cognitive impairment. However, the cumulative exposure time of HCP to SARS-CoV-2 would increase the risk of transmission [National Center for Immunization and Respiratory Diseases (NCIRD), 2021].

After further mobile optimisation, our methodology might also expand in-home application scenarios with the rapid growth of smart device owners, globally (Silver and Taylor, 2019). With the rapid and large-scale growth of the elderly population in the world, there is a huge gap between the supply and demand of health monitoring and disease screening. Solutions aimed at reducing the strain on elderly care facilities and promoting independence, such as technology-enabled home-care services, will become the major part of the elderly care model in the near future (Mesko et al., 2018). Machine vision with artificial neural network tools has produced opportunities for convenient at-home screening of geriatric diseases such as frailty (Ahmed et al., 2020).

## Data Availability Statement

The raw data supporting the conclusions of this article will be made available by the authors, without undue reservation.

## Ethics Statement

The studies involving human participants were reviewed and approved by the Biomedical Ethics Committee of West China Hospital, Sichuan University. The patients/participants provided their written informed consent to participate in this study. Written informed consent was obtained from the individual(s) for the publication of any potentially identifiable images or data included in this article.

## Author Contributions

YL contributed to the design and carried out the current study. RW and YD contributed to modification of the experimental protocol. RH and QT contributed to optimisation of the silhouette segmentation module. XH and LQ contributed to key point extraction. BY and ZW contributed to the feature extraction module. YM, XLi, HW, XLiu, LZ, LD, ZX, and CX contributed to the film and edited the gait video. MG, XS, JJ, JC, XLin, and LX were involved in baseline information gathering. HG and HY analysed the experimental data. BD drafted the manuscript. XHH supervised the current research. All authors contributed to the article and approved the submitted version.

## Conflict of Interest

XLi is employed by Aviation Industry Corporation of China 363 Hospital. The remaining authors declare that the research was conducted in the absence of any commercial or financial relationships that could be construed as a potential conflict of interest.

## Publisher’s Note

All claims expressed in this article are solely those of the authors and do not necessarily represent those of their affiliated organizations, or those of the publisher, the editors and the reviewers. Any product that may be evaluated in this article, or claim that may be made by its manufacturer, is not guaranteed or endorsed by the publisher.
