# Supplementary material for: Application of Machine Vision in Classifying Gait Frailty Among Older Adults
Source: Front Aging Neurosci. 2021 Nov 16;13:757823. doi: 10.3389/fnagi.2021.757823 (PMC8637841; doi:10.3389/fnagi.2021.757823)
Supplement: Supplementary file 1 [file Data_Sheet_1.docx]

**Supplementary** **Material**

**Supplementary Section 1. Statistical Analysis**

The Data were summarized as the mean ± standard deviation. The normality test was realized when the number of samples was sufficient (Shapiro-Wilk test). If samples followed a Gaussian distribution. One-way analysis of variance (ANOVA) was used to compare the differences between groups. When the samples failed the normality test, a non-parametric test (Mann-Whitney test) was applied. A *P*-value which was less than 0.05 was used as a cut-off to define statistical significance. Overall accuracy of machine vision classifier, AUC of the ROC and Kappa coefficients were used to evaluate the performance of machine vision classifiers. Firstly, the probability matrix output via full-trained AlexNet and VGG16 after a softmax transition was analysed through RStudio version 4.0.2 of the MultiROC package (Supplementary Table2). Then, the ROC curves were plotted by GraphPad 8.0.1. The Kappa coefficients for FFP and machine vision classifier predictive results were calculated via SPSS 19.0.0.

Furthermore, the three-column predictive probability matrix output from AlexNet and VGG16 was transferred to three probability columns as three two-classification tasks (healthy or unhealthy, etc). Similarly, other potential predictive parameters (age, time of 4m walking, and grip strength) of each participant were indexed with three pairs of two-classification-label respectively. Then, two-classification AUCs of ROC were calculated via GraphPad. The two-classification sensitivity, specificity, positive predictive value (PPV), and negative predictive value (NPV) for AlexNet and VGG16 compared with ground truth were calculated via SPSS.

**Supplementary Section 2. Alphapose**

Alphapose framework (H. Fang, 2017), a multi-person pose estimation model, is based on previous two-step framework, which first detects the bounding box of human body, and then estimates the pose of each box independently. Firstly, a VGG-based single shot multi box detector (SSD) module (Wei Liu, 2016) was applied as a human detector in Alphapose. In order to guarantee the extraction of the entire person region, the detected human proposals are extended by 30% in both the height and width directions. The spatial transformer network (STN) was trained to perform a 2D affine transformation to image. After extracting high-quality dominant human proposal regions, the single person pose estimator (SPPE) (Newell A., 2016) could be utilized for accurate pose estimation. Naturally, a spatial de-transformer network (SDTN) is required to remap the estimated human pose back to the original image coordinates. Finally, the parametric pose non-maximum-suppression (Pose NMS) is carried out to eliminate redundant pose estimations.

**Reference**

H. Fang, S.X., Y. Tai and C. Lu (2017). RMPE: Regional Multi-person Pose Estimation. *2017 IEEE International Conference on Computer Vision (ICCV), Venice*. doi: 10.1109/ICCV.2017.256.

Newell A., Y.K., Deng J (2016). "Stacked Hourglass Networks for Human Pose Estimation", in: *ECCV 2016 Lecture Notes in Computer Science.* Springer).

Wei Liu, D.A., Dumitru Erhan, Christian Szegedy, Scott Reed, Cheng-Yang Fu, Alexander C. Berg (2016). "SSD: Single Shot MultiBox Detector", in: *ECCV 2016: Computer Vision.*).

**Supplementary Table 1. Criteria of Physical Frailty Phenotype (Based on Definition of Fried)**

|  | Frailty phenotype | Measurement |
| --- | --- | --- |
|  | Shrinking | Weight loss of 4.5kg in the last 12 months or body mass index (BMI) <18.5 kg/m^2^ |
|  | Slowness | Usual-pace 4-meters walking trials, ≤25th percentile within four sex-by height categories  **Male:** Height ≤162.0 cm, >6.1s; Height >162.0 cm, >5.8s  **Female:** Height≤150.8cm, >6.9s; Height >150.8 cm, >6.1s |
|  | Weakness | Maximum grip strength of dominant hand over 2 trials≤25th percentile within eight sex by–BMI categories  **Male:** BMI≥28, <31.0Kg; 28>BMI≥24, <29.9Kg; 24>BMI≥18.5, <26.5Kg; BMI<18.5, <25.5Kg  **Female:** BMI≥28, <19.2Kg; 28>BMI≥24, <19.1Kg; 24>BMI≥18.5, <18.3Kg; BMI<18.5, <15.3Kg |
|  | Exhaustion | Participants self-reported unusually fatigue or weak at a moderate amount of time (3-4 days per week) or most of the time (5-7 days per week);  Or self-report low energy (using the numerical rating scale to assess the energy level from 0 to 10, where 0 represents the lowest energy and 10 represents the most energy, low energy defined if score <3). |
|  | Low physical activity | CLTPAQ≤25th percentile within four sex-by-total energy expenditure per week;  Energy consumption (kcal /week) =MET× frequency (per week) × time (minutes) ×body mass (Kg)/60;  Categories of physical activities included walking (4.0 MET), indoor housework (3.5 MET), outdoor housework (5.0 MET), dancing (4.5 MET), playing ping-pong (4.0 MET) and other regular exercises (5.0 MET).  **Male:** Height ≤162.0 cm, < 2010Kal/week; Height >162.0 cm, < 2334Kal/week  **Female:** Height≤150.8cm, <1626Kal/week; Height >150.8 cm, <2047Kal/week |

**Supplementary Table 2. Download Location of Resource**

| Pre-trained AlphaPose:  https://drive.google.com/file/d/1nxyfUbvWDaaT9eDM7Y31ScSVPlGy6gfw/view |
| --- |
| COCO and OCHuman:  https://github.com/liruilong940607/Pose2Seg |
| Pre-trainedPose2Seg: https://drive.google.com/file/d/193i8b40MJFxawcJoNLq1sG0vhAeLoVJG/view |
| Pre-trained Gaitset:  https://onedrive.live.com/?authkey=%21AHx2vMi_-mFNHmY&id=50DC29123BD9D3EA%217395&cid=50DC29123BD9D3EA |
| Pre-trained VGG and AlexNet model:  https://pytorch.org/vision/stable/models.html |
| MultiROC package:  https://github.com/WandeRum/multiROC#multiroc- |

**Supplementary Table 3. Evaluation of Segmentation Methods in Open-Source Dataset.**

| Model/Data set | COCO | | OCHuman | |
| --- | --- | --- | --- | --- |
|  | mAP | AP(large) | mAP | AP(large) |
| Mask R-CNN | 0.532 | 0.638 | 0.163 | 0.113 |
| Pose2Seg | 0.557 | 0.661 | 0.507 | 0.512 |
| DPose2Seg | 0.577 | 0.682 | 0.547 | 0.550 |

**Supplementary Table 4. Computational Time Consuming for Different Machine Vision Analysis Task**

| Stage of Analysis | Model | Time-consuming in training and test* | Time-consuming in generalization for one gait record |
| --- | --- | --- | --- |
| Body Key Points Extraction | Alphapose | ---- | 10 seconds |
| Silhouettes Segmentation | Pose2Seg | 7 days | 10 seconds |
|  | DPose2Seg | 9 days | 10 seconds |
| Gait Features Extraction | Gait-set | 4 days 3 hours | ---- |
|  | LGaitset | 5 days 4 hours | ---- |
|  | DGaitset | 6 days | ---- |
|  | Customised CAE** | ---- | 15 seconds |
| Identification of Gait Features | VGG16 | 5 days | 3 minutes |
|  | AlexNet | 5 days | 3 minutes |

*The detail of data assembly were present in method part (2.4.2-2.4.4) and supplementary figure 1, 2; **The GPU for all task was an NVIDIA GeForce GTX1080Ti graphics card and the operating system used was Ubuntu 16.04.


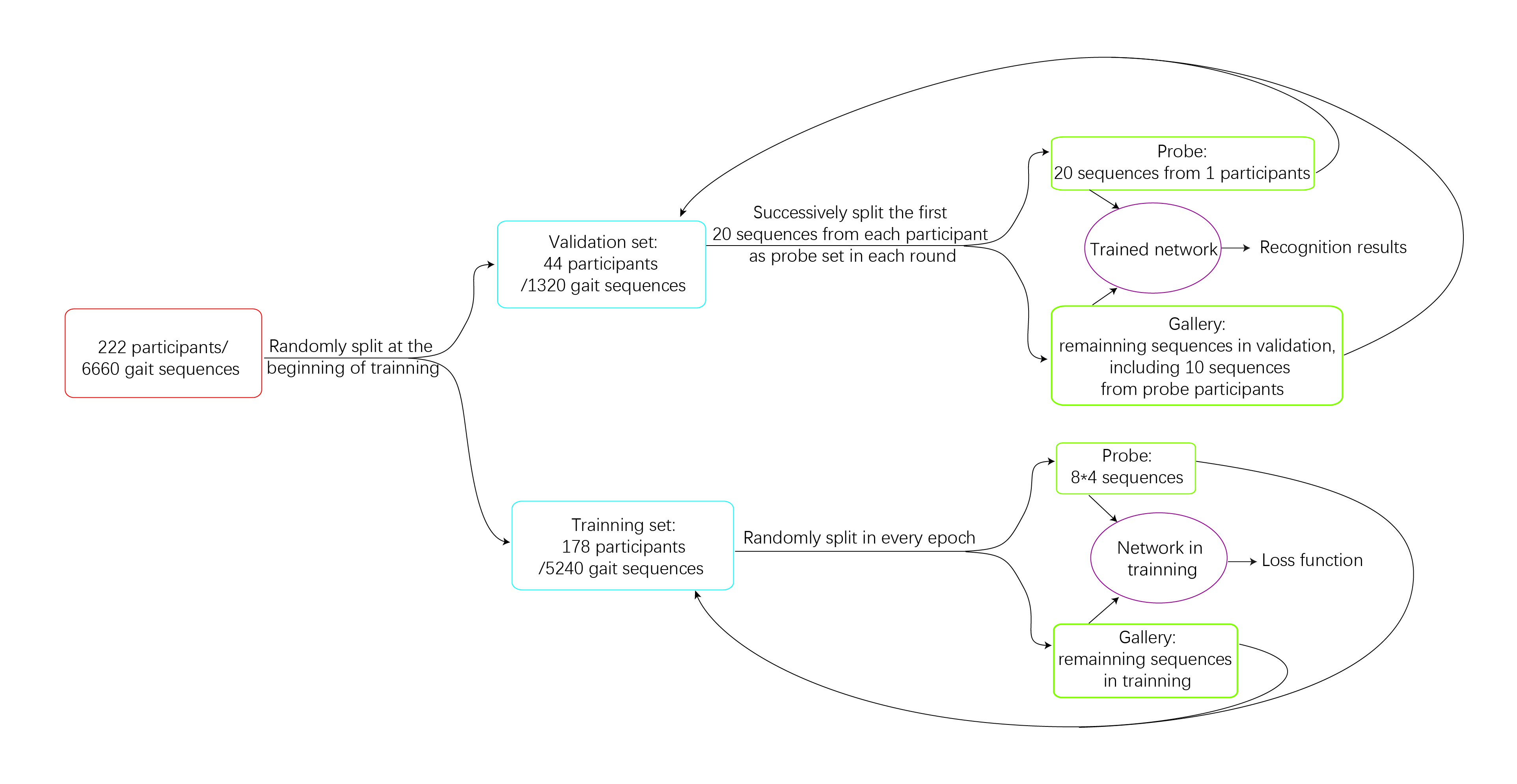


**Supplementary Figure 1. Detail of data assembly and analysing in recognition task**

**Supplementary Figure 2. Detail of data assembly and analysing in classification task**
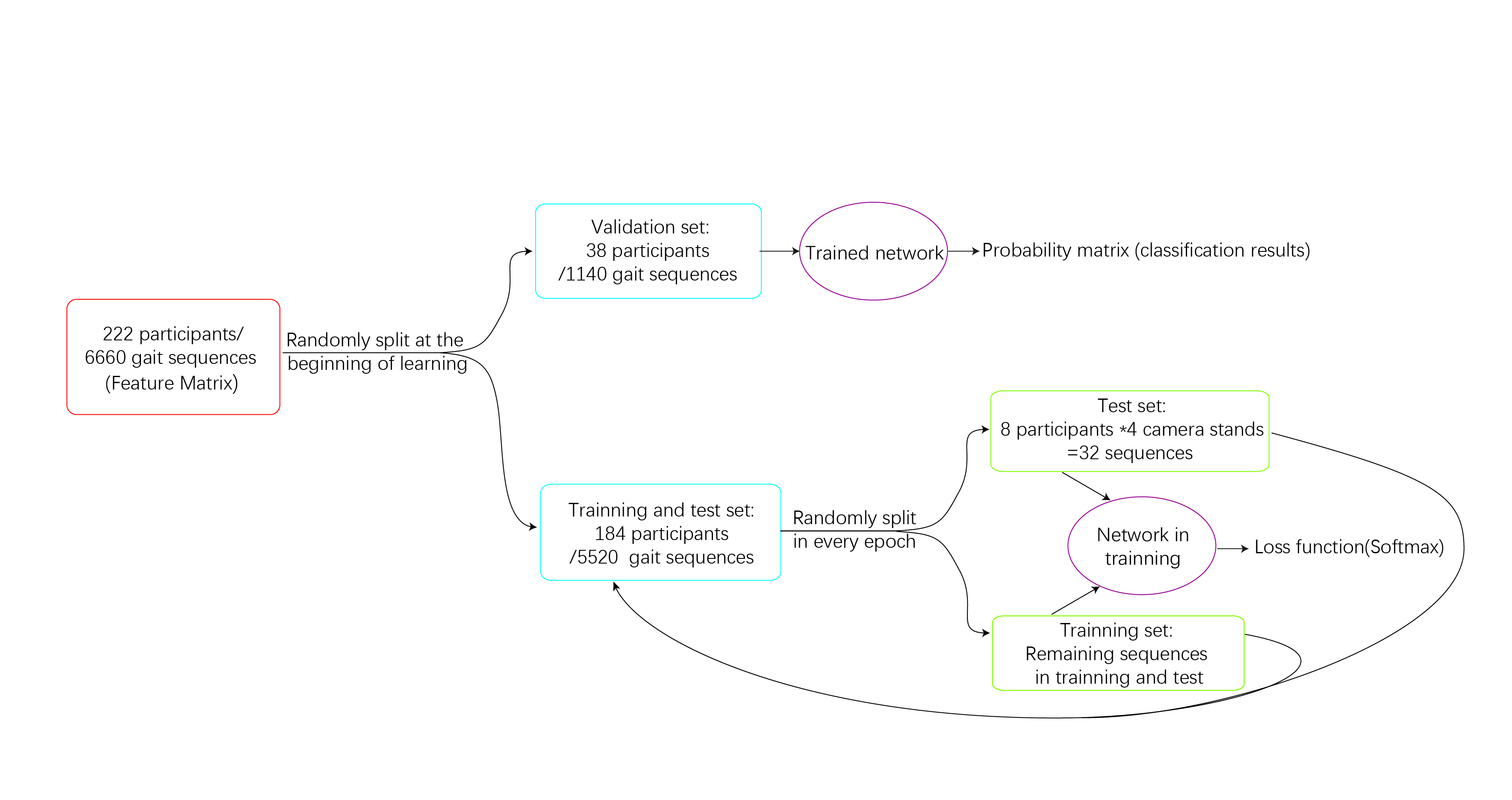


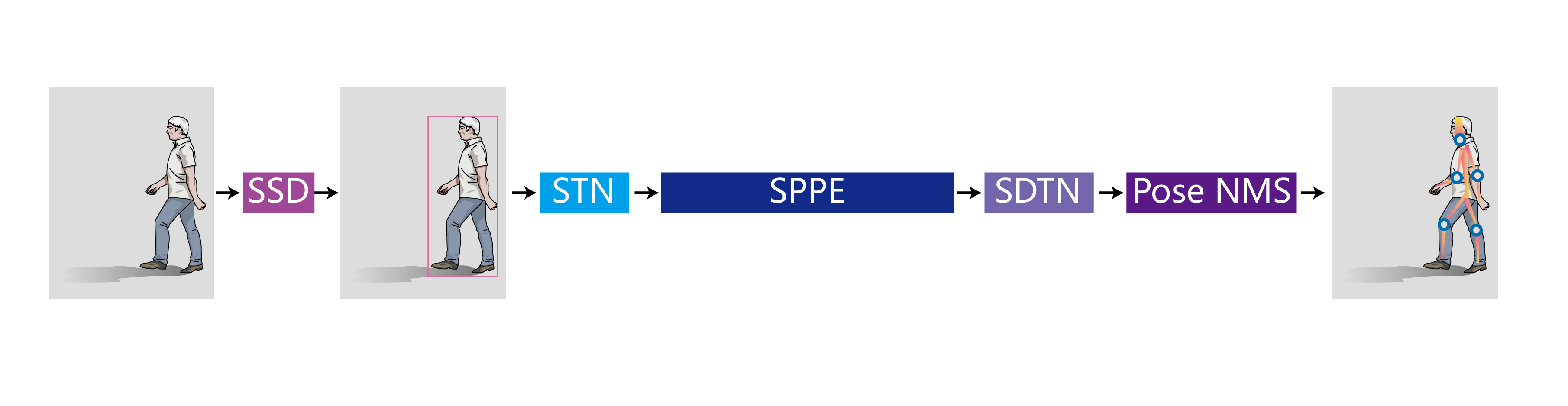


**Supplementary Figure 3. Alphapose Framework**

SSD: single shot multi box detector; STN: spatial transformer network; SPPE: single person pose estimator; SDTN: spatial de-transformer network; Pose NMS: pose non-maximum-suppression
